# Unilateral Cleft lip and Palate: Long-Term Results of the Malek Technique

**DOI:** 10.1177/10556656221139671

**Published:** 2022-12-01

**Authors:** Anthony de Buys Roessingh, Yohann Robert, Josée Despars, Chantal Zbinden-Trichet, Georges Herzog, Martin Broome, Oumama El Ezzi

**Affiliations:** 1 Department of Pediatric Surgery, University Hospital Center of the Canton of Vaud (CHUV), Lausanne, Switzerland; 2 Multidisciplinary consultations of facial cleft, CHUV, Lausanne, Switzerland

**Keywords:** pediatrics, surgical technique, speech development

## Abstract

**Objective:**

To review at 18 years-old the results of surgery and follow-up of children born in our hospital with unilateral cleft lip and palate (uCLP). They were operated at the time by the same surgeon, following the same primary surgical procedure (Malek).

**Design:**

Retrospective cohort study.

**Setting:**

Tertiary Children's Hospital.

**Patients:**

All children born with uCLP between 1996 and 2001 and operated in our hospital. Syndromic children were excluded.

**Main outcome measures:**

Results of the primary surgery, ear–nose–throat interventions, maxillo-facial surgery and final phonatory results.

**Results:**

Seventy-nine files of children born with a cleft were reviewed: 34 were taken into consideration for uCLP: 15 right and 19 left. They were operated in two stages, following the inverse Malek procedure. Sixty per cent had a fistula. Eighty-eight percent had grommets. Ninety-seven percent had an alveolar graft at a median age of nine (5-10) and 22% underwent a Le Fort osteotomy. Seven percent were operated for a pharyngeal flap, 29% for a secondary lip surgery at a mean age of 12.8 and 29% for a late rhinoplasty at a mean age of 14.8 years. A median of 5.7 multidisciplinary consultations was realized with a median number of general anesthesia of 7.1 (4-13).

**Conclusions:**

This retrospective study shows that the Malek procedure for children born with uCLP is related to a high risk of fistula but good long-term phonatory results. Twenty percent of children were operated for a Le Fort procedure and one-third for a secondary lip procedure and rhinoplasty.

## Introduction

A facial cleft can be labial, labio-maxillary (unilateral or bilateral), labio-maxillary-palatal (unilateral or bilateral), or isolated palatal.^1^ Depending on the type of cleft and the age of the child, feeding, speech, ear-nose-throat (ENT), dental, orthodontic, aesthetic, and psychological problems will be present.^2,3^

Unilateral cleft of the lip and palate (uCLP) is the most common type and represents 37% to 50% of all cases of clefts.^4,5^ Clefts are twice as frequent in Asia as in Europe, and half as frequent among the black population. Their incidence seems to be higher in poor social conditions.^6^

In developed countries, handling of a cleft starts at the time it is diagnosed, ideally before birth, and ends when the child is fully grown.^7^ A multidisciplinary team of experienced specialists is a necessity in order to handle the diverse problems of children born with a cleft and to follow them through each developmental stage. The objective of the team is to bring together specialists in rehabilitation in order to optimize the care and the treatment in all fields: facial aesthetics, orthodontics, ENT, speech, and psychological.^8^

The ideal age for surgical cleft repair remains a subject of debate. Thanks to the progress in pediatric anesthesia, surgery can be performed at a very early age. The timing and sequence of primary surgery may vary largely, depending on the local philosophy and history of surgery. The inverse Malek procedure for uCLP is soft palate repair at the age of 3 months and hard palate combined with lip closure at five months of age.^2^ For uCLP, a vomer-flap may be used to reconstruct the nasal layer of the velum.

The sequelae of facial clefts are numerous and involve many tissues, organs, and systems: lip and nose from an aesthetic point of view, and long-term breathing and speaking from a functional point of view. The impact on the personality of the child and his psychological well-being must also be taken into account.^9^ Therefore, cleft treatment starts at the time it is diagnosed and ends when the child is fully grown. The success of cleft repair should not be measured solely on the basis of a good esthetic result, but also, in the long run, on whether it facilitates the social acceptance of the child, his possibility to attend school and receive an education, and his integration in the social activities, all of which initially depend to a large extent on the speech quality resulting from the operation, speech being a cornerstone of social integration.^10^

The aim of this retrospective study is to report the long-term results of the multi-disciplinary care of patients born with a uCLP, treated by one surgeon using the same primary surgical technique and timing.

## Methods

This project was conducted under the authorization of the local Ethics committee (CER-VAUD), decision 2019-00468. Each participant received a phone-call with an oral explanation in order to be included in the study. The different demographic data acquired were gender, precise type of malformation, and association with any syndrome. Operative data were subdivided into plastic, ENT, and maxillofacial procedures. Plastic pediatric interventions included veloplasty, labioplasty, palatoplasty, closure of oro-nasal fistula, and pharyngeal flap. Orthophonic and psychological follow-up were noted. All data were recorded from files of children on a computer (Soarian^®^, computerized medical charts of the pediatric surgery department, CHUV, Lausanne) and from written files. Excel sheet using codification with a neutral number specific to the study was used. All numeric data were protected with a password and all paper data were kept in a key-closed drawer.

We identified all children born with uCLP between 1996 and 2001. Children born with associated malformations or chromosomal abnormalities were excluded from the study; incomplete files were excluded; partial uCLP was excluded; children from our surgical activity in Africa were also excluded. All children born with clefts were fitted with a removable palatal appliance before 1 week of age and were bottle fed with a normal teat and expressed breast milk. A pediatrician followed the children to evaluate their psychomotor development.

All children were operated by the same surgeon, chief of the department and specialist in plastic surgery in children, especially in cleft repair.

### Organization

Our multidisciplinary cleft team was composed of the pediatric surgeon, two pediatric ENT specialists, a craniofacial surgeon, an orthodontist, two speech therapists, and a psychologist. A geneticist and a gynecologist are also present to offer their collaboration to the discussion.

The multidisciplinary cleft team works 1 to 2 days per month. Fifteen children and their parents are evaluated alone, the same morning by each of the members of the team. In the afternoon, the members of the multidisciplinary cleft team are in one room, all together to see the same 15 children (and their parents) one by one for a final explanation and for organizing the follow-up. The child and its parents must be seen as required by the child's and the parents’ needs. For example, maxillofacial surgery is correlated with orthodontic treatment; in our team, it is performed at the end of the growth period, between the ages of 16 and 18. The craniofacial surgeon, always present in the team, does all the bone grafts and the maxillary advancements. The pediatric plastic surgeon is the team-leader in our multidisciplinary team: he does the primary and the secondary surgery excepting the rhinoplasty and coordinates the follow-up of the children. The child and its parents may be seen by the specialists alone, many times without the presence of the team, depending on the child's needs.

Since 1990, soft palate repair on children born with a complete uCLP is performed at the age of 3 months and hard palate and lip closure at 5 months. A vomer-flap is used to reconstruct the nasal layer of the velum if necessary. The pediatric surgeon was the team-leader in our multidisciplinary team. She did all the primary and secondary surgery (except the rhinoplasty) and coordinated the follow-up of all children.

All children were assessed by the pediatric surgeon before and after surgery, and then 6 months later, and once a year until the age of 3.5, when they were evaluated by a multidisciplinary team. When the child was 1 year old, its parents were taught strategies to encourage babbling and early verbal communication. The ENT specialist checked hearing once a year. Pediatricians were instructed in the special care needed to diagnose chronic serous otitis media and its risk of hearing loss in patients born with a cleft. They were asked to perform routine otoscopy and tympanometry and to check for glue ear.

### Primary Operative Technique

A palatal plate made of acrylic resin, with a rigid outer layer and a softer inner layer, was made by the orthodontist usually on the day after birth, at the hospital. Veloplasty was the first operation, done according to the inverse Malek procedure at 3 months. The child was installed at one end of the table. The head rested on an adjustable support with a hyperextension position. A pad placed under the shoulders kept the neck extended. The surgeon stood at the end of the table, with the assistant on the right side of the table and the anesthesiologist on the left side. The infant's eyes were taped shut with sterile strips with a drop of vaseline. The surgical equipment included the specific instruments required for palate surgery.

**Figure 1.**
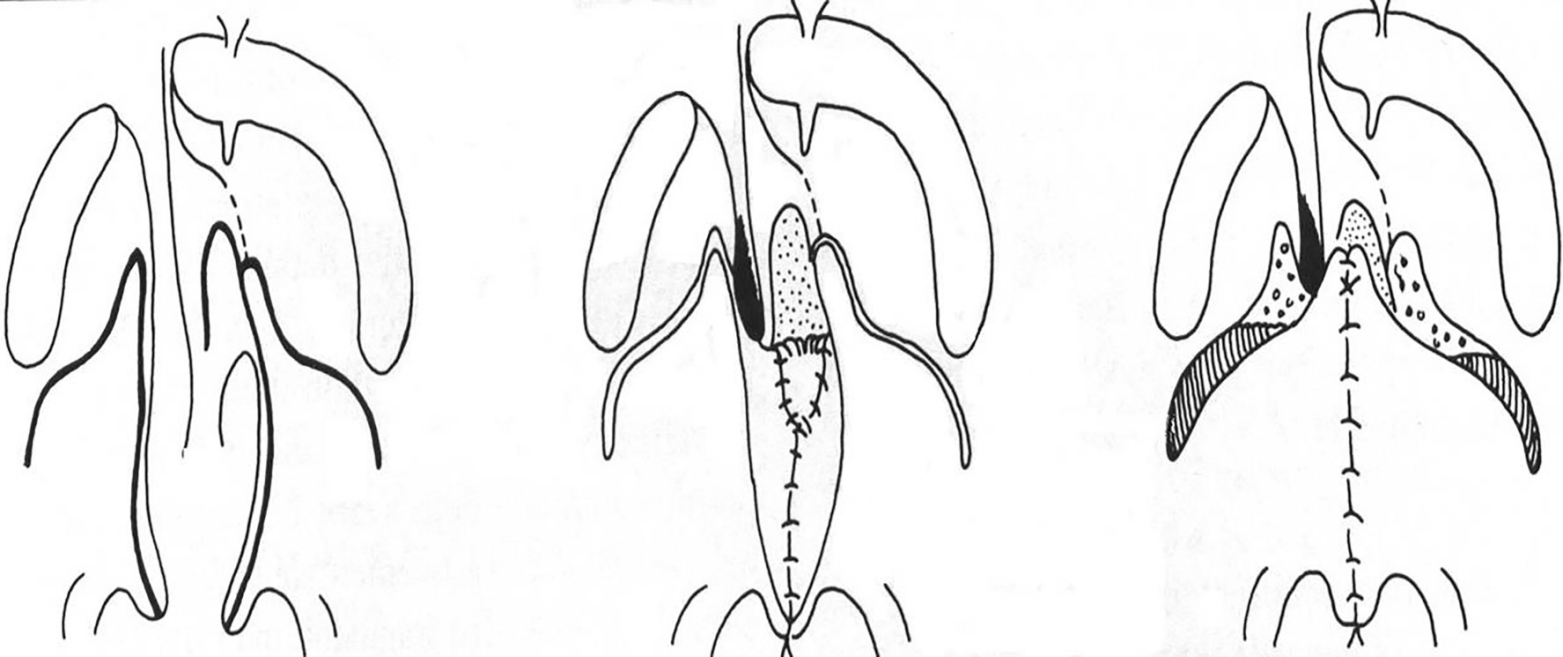
Schematic description of the veloplasty.^2^

Incisions were made along the margins of the cleft as far as the uvula in order to create two flaps. The levator veli palatini muscles are part of the superior velar muscles and are oriented in the same direction as the Eustachian tube. In a cleft palate, they do not join on the midline. Their fibers run parallel to the margins of the cleft and have therefore lost their original transverse orientation. Both hamuli were then broken with a Trélat elevator in order to relax the tendon of the tensor veli-palatini. Muscle attachments to the palatal shelf were sectioned behind the neurovascular bundle. One then proceeded to the undermining, with the tissues being pushed back and carefully divided until the detachment of the soft palate was completed. The half-velum was thus completely detached from the bone and could be drawn inward for later suturing. The muscle fibers were carefully attached together with a horizontal orientation. This procedure created nasal and oral layers (Figure 1). The first layer of the nasal mucosa was sutured with 5-0 or 4-0 absorbable filament (Vicryl^©^ 5.0). For the veloplasty, when direct suturing was not possible, it became necessary to create a nasal layer using vomerine mucosa, a procedure practiced by Veau^11^ and later by Petit. A longitudinal incision was made in the lower edge of the vomerine mucosa and nasal mucosa, on either side, from front to back, and was then sutured. The naso-vomerine suture continued until the tension appeared to diminish and then, at this point, direct suturing of the nasal mucosa could be performed.^2^

**Figure 2.**
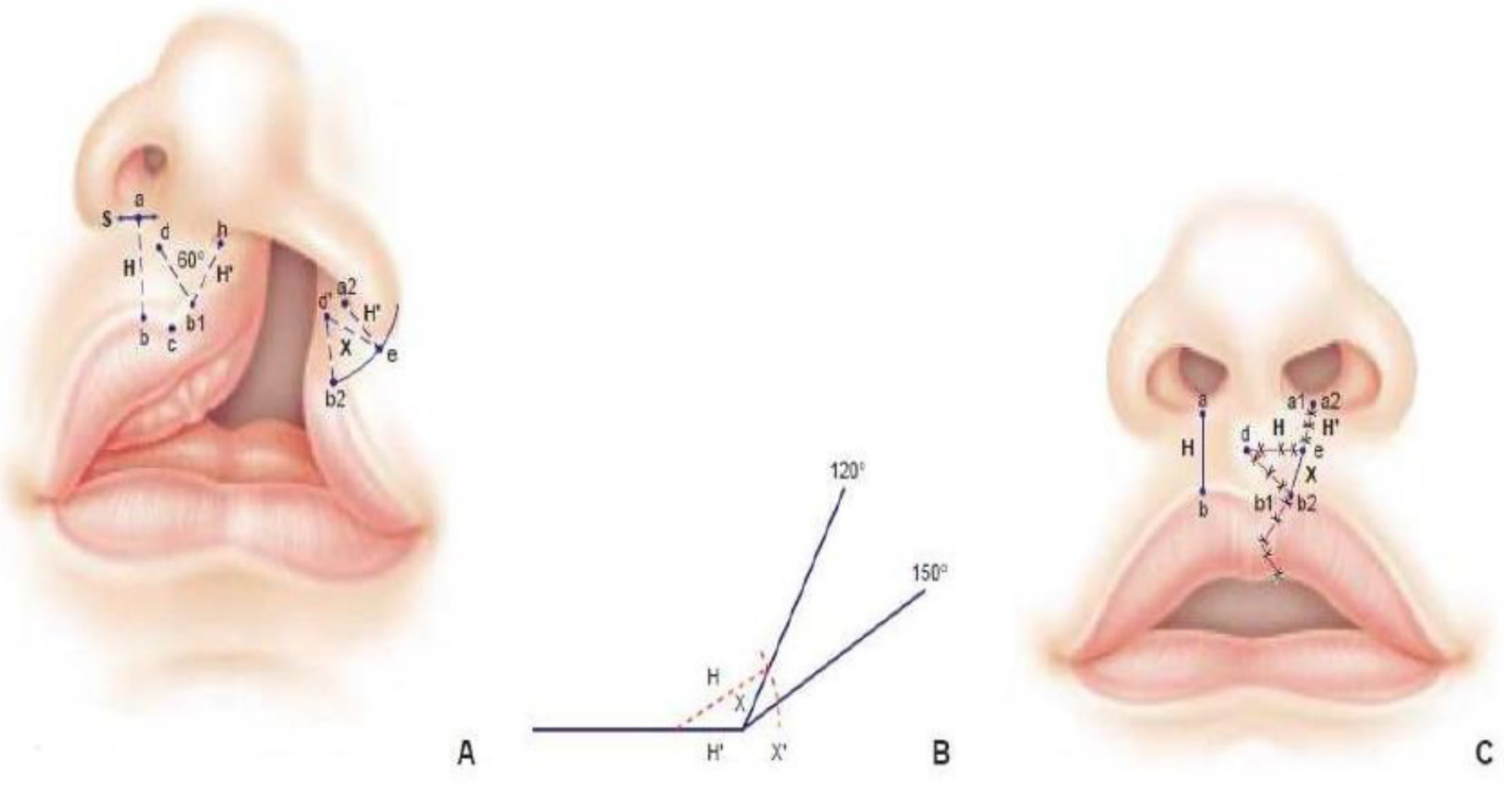
Labioplasty was realized following the inverse Malek procedure^2^ at 5 months of age in order to create 2 isoceles triangle flaps with incisions in both border.

Labioplasty was performed following the inverse Malek procedure at 5 months of age. The aim was to create 2 isocele triangle flaps with incisions on both sides. This procedure was based on the principle of a Z-plasty (Figure 2). Surgery started with the drawing of the lip repair. Then the tissues were usually infiltrated with Bupivacaine hydrochlorate-adrenaline in order to facilitate the mucoperiostal undermining for lip repair and to reduce bleeding thanks to the temporary hemostatic effect of the vasoconstrictor. The exact symmetry between the two sides was calculated with mathematical precision. Skin without muscle was eliminated. The muscle for both sides was sutured together knowing that the orientation of the muscle fibers depends on the outlines of the incision, since the cutaneous-muscular flaps are used with a certain degree of rotation. The incision took into account the final direction of the muscle fibers which was reinserted.

**Figure 3.**
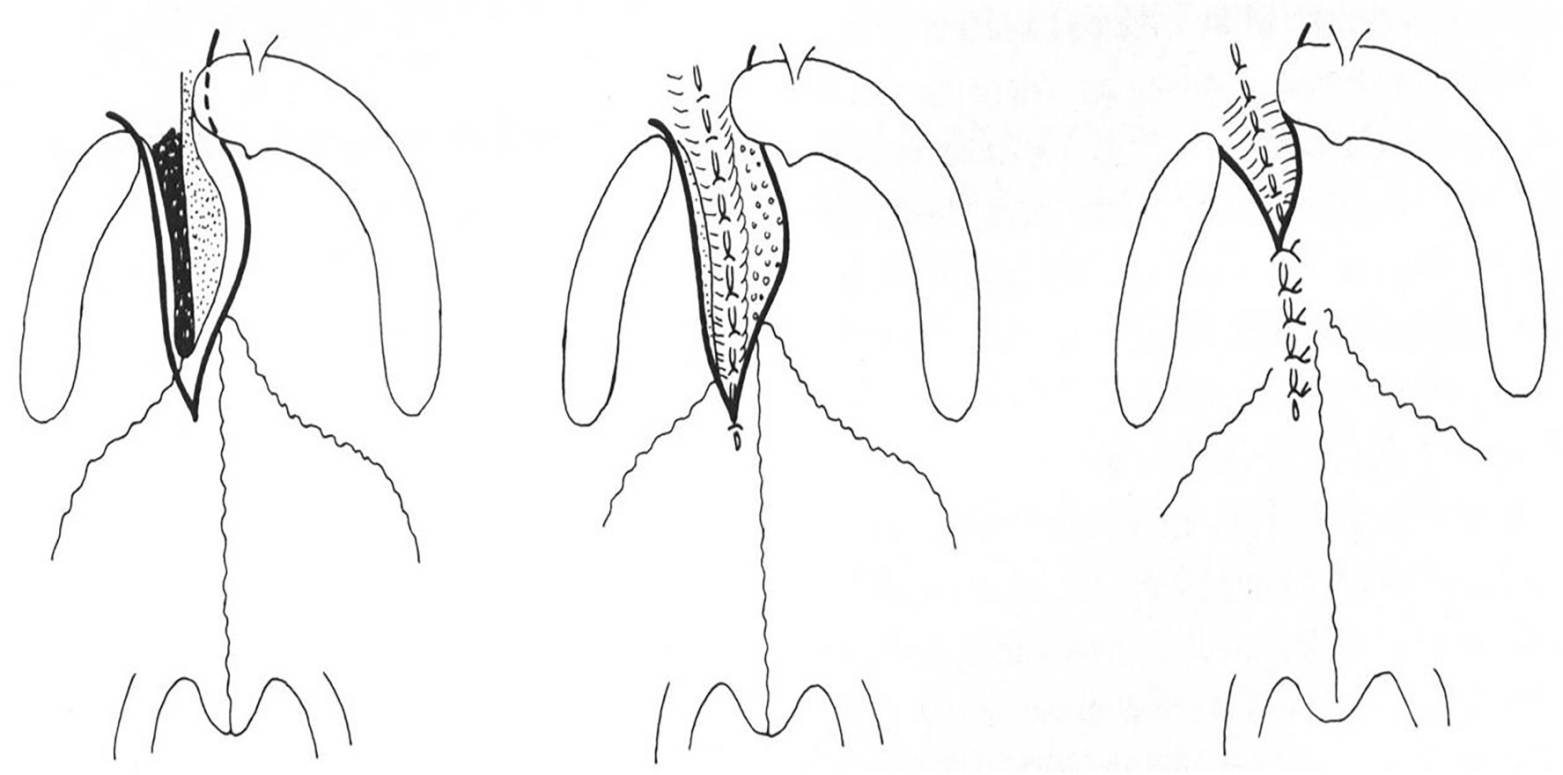
Schematic description of the palatal closure.^2^

The technique of palatoplasty at 5 months included a longitudinal incision that divided the border of the cleft and continued forward up to the apex of the cleft, which often extended into the bony vault. Two flaps without incision behind the maxillary tuberosity or alveolar arch were simply done. No extensive dissection was done (Figure 3).

### Hearing

In cases of glue ear visualized under microscope after a myringotomy, grommets were inserted at the time of the palatoplasty, under the same anesthesia.^12^ Hearing assessment was performed at variable ages and in response to individual needs. It was performed in a reproducible manner, with impedance tympanograms using a Grason-Stadler GSI-28A tympanometer, and with total audiograms using a Grason-Stadler GSI-16 earphone audiometer (Grason-Stadler, Littleton, MA, USA). These completed the ENT's clinical examination and were performed on the same day as the speech evaluation.

Our clinical indications to perform tympanostomy and insert grommets were chronic episodes of otitis media effusion (OME), tympanic membrane retraction, perforation, or abnormal tympanometry/audiometry with a hearing loss of more than 30 dB in one ear. The parents had reported a hearing loss and disorder in speech acquisition. OME was considered persistent if it lasted more than 3 months, especially after the summer. We put short-term grommets in gold or metal under microscope viewing. The insertions were realized with a microsurgical knife, in the lower internal quadrant. Follow-up was done by the same pediatric otolaryngologist. Grommets removal was realized either spontaneously or after routine controls. The children were then seen 3 months later to check the anatomy. Indications and time of extraction were based on clinical examination and improvement of hearing of each child.

### Speech

Speech evaluation was performed at the age of 3 and, subsequently, whenever it was deemed necessary. Perceptual speech evaluation by qualified and experienced speech pathologists is the mainstay of speech evaluation in our institution. The individual evaluations of all patients, made separately by 2 therapists, were discussed and compared in an attempt to minimize listener's bias. The children were interviewed in a quiet playroom in the presence of a parent. Standard upper airway assessments were documented, including the presence or absence of snoring, mouth breathing, nasal airway obstruction, and sleep apnea. Velo-pharyngeal insufficiency (VPI) or nasal air emission was evaluated according to the Borel-Maisonny classification (Table 1).^13,14^ Hyper-nasality, hypo-nasality, audible nasal emission, voice quality, misarticulations associated with VPI, and intelligibility were assessed. Nasal emission on separate phonemes was measured using a 622 Kay Electronics nasometer (Kay Elemetrics, Pine Brook, NJ, USA). Fluoroscopic velopharyngeal evaluations were not considered. The same two therapists performed clinical perceptual speech evaluations and nasometry before and after operations. The presence of nasal phonation led to automatic classification as a type 2 phonation score.^15^ Articulation errors were divided into categories based on anatomical origin and backing, stops and fricative sounds were recorded for each anatomical region, as were compensatory articulations (Table 2).

**Table 1.** Borel-Maisonny Classification.

Type O	No phonation
Type 1	Excellent phonation, no nasal air emission
Type ½	Good phonation, intermittent nasal air emission, good intelligibility
Type 2	Phonation with continuous nasal emission
Type 2b	Phonation with continuous nasal emission but good intelligibility and no social discomfort
Type 2M	Phonation with continuous nasal emission, bad intelligibility
Type 2/3	Phonation with continuous nasal emission with compensatory articulation, bad intelligibility

**Table 2.** Compensatory Phenomena Related or not Related to Velopharyngeal Insufficiency (VPI).

Simple misarticulation, not related to VPI	Heavy misarticualtion	Voice trouble	Compensatory movements	Added sounds
Sigmatisms	Articualtions compensation	Hpo-nasality	Facial (syncinesia)	Snoring
Posterioriations	Glottic sounds	Hyper-nasality		Mouth breathing
Deletion of consonants	Raucity	Raucity		Clics
Confusions Fricative sounds Confusions oral nasal Backing				

### Pharyngeal Flaps

**Figure 4.**
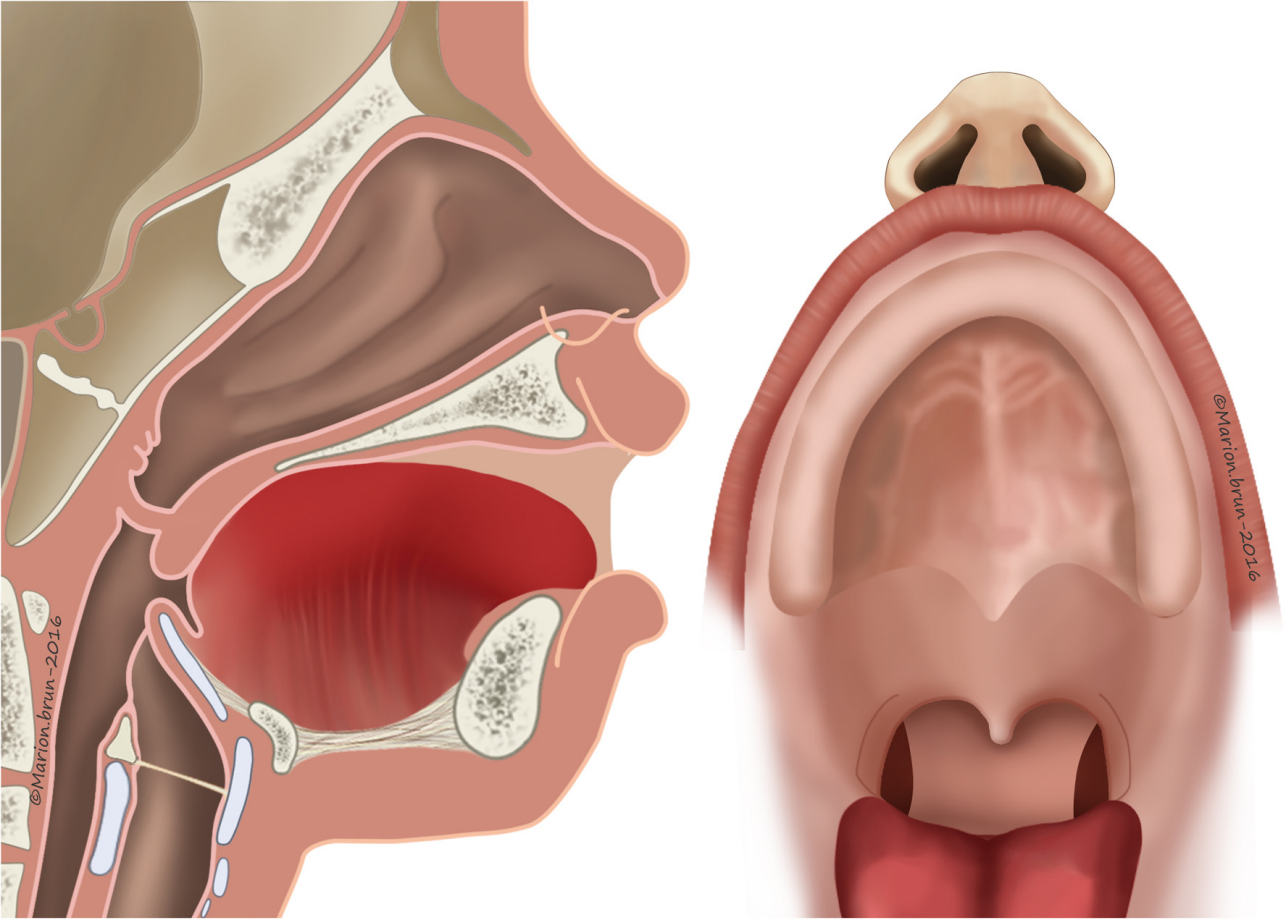
Cranial-based pharyngeal flaps were performed according to Schönborn^19^ and Sanvenero-Rosselli^17^ modified by Fischer Brandies^14,18^

The criteria for recommending pharyngeal flap surgery were based on perceptual analysis: hypernasality, weak pressure consonants, weak pharyngeal musculature, and nasal emission (type 2 M, 2/3 Borel-Maisonny). Cranial-based pharyngeal flaps (Figure 4) were performed according to Schönborn and Sanvenero-Rosselli.^16–18^ A broad, cranially based pharyngeal flap was incised and elevated from the prevertebral fascia to be sutured to the nasal side of the incised velum. We tried to do a cranially based pharyngeal flap as large as possible in order to close the donor site directly and to have a large flap knowing that over the following months this flap will shrink.

Velopharyngeal assessment was performed 6 and 12 months after surgery. Surgical success was defined in terms of elimination of perceptible hypernasality or oral resonance and instrumental evidence of complete velopharyngeal closure by nasoendoscopy. Surgical failure was defined in terms of persistent hypernasality and/or nasal turbulence observed in a perceptual speech evaluation and of incomplete velopharyngeal closure evidenced by nasometry 6 months at least after surgery. Evaluation to determine the presence of sleep apnea was done at 4 months after surgery and then again after 1 year (symptoms including fitful sleep, unresolved snoring, and daytime fatigue).

### Orthodontic Treatment and Alveolar Graft

Malposition of the teeth was not usually treated during the mixed dentition period, because of its transitory nature at this stage. Before this period, parents were advised to control the application of the necessary dental hygiene. In the presence of a cleft of the alveolus with dental agenesis (upper lateral incisor), or hypoplastic/malpositioned teeth, orthodontic treatment was correlated with the planning of the alveolar bone graft.^20,21^ The preparation of the alveolar bone graft was begun before the age of 9 or 10. In order to normalize the shape of the dental arch, orthodontic treatment was performed by using an asymmetrical expansion plate followed by a period of retention; after the graft, the anterior teeth were aligned with fixed orthodontic appliances, and later stabilized with a retainer made of twisted steel wire, bonded to the lingual side of the teeth with composite resin.

Cancellous bone for the graft was harvested, usually from the iliac crest or from the mandibular ramus.^22^ Orthodontic diagnosis and treatment planning were done around the age of 12, that is, by the end of the mixed dentition period. Orthodontic treatment was often combined with orthognathic surgery.

### Orthognathic Surgery

Orthognathic surgery to correct facial disharmony was part of the normal follow-up in our institution for children born with uCLP. When maxillary growth deficiency was obvious, it was confirmed by an analysis of cephalometric measurements. These measurements were made regularly, because bone growth of individual parts could happen at different speeds, and a good articular congruence with a good esthetic profile could deteriorate quite quickly during puberty. When planning corrective surgery, facial aesthetics, profile harmony and intermaxillary occlusal relationships are essential parameters to consider. Objective indications for orthognathic surgery are based on cephalometric variables: intermaxillary relationship in the sagittal plane: SNA, SNB, and ANB angles (S = sella, N = nasion, A = subspinal, and B = supramental) and Wits appraisal (perpendiculars from points A and B onto the occlusal plane). The distance from the upper lip to the e-Line (line drawn from the tip of the nose to the chin) is one of the measurements used to assess soft tissue profile harmony. A maxillary advancement with a Le Fort I osteotomy was the most common orthognathic procedure. Due to a possible transversal collapse of the maxillary arches on each side of the cleft, caused by the scarring tissue, this advancement could not always be achieved in one piece. In these cases, the maxilla needed to be segmented into 2 or 3 pieces. Surgical treatment was correlated with the orthodontic treatment and performed at the end of the growth period, between the ages of 16 and 18. The cranio-facial surgeon, always present in the team, did all the bone grafts and the maxillary advancements.

### Rhinoplasty

Ideally, rhinoplasty was planned at the end of growth and after completion of orthodontic treatment. The operation involved a small incision below the nose and necessitated a cartilage graft harvested from the ear. The deviation of the nasal septum was also corrected during the same surgical procedure. A thermoformable splint was used for several days or weeks. This surgery represented the final step in the mutlidisciplinary follow-up.

### Psychological Support

Psychological support was offered to the parents from the time when prenatal diagnosis was established and as long as this support was required and needed. Prenatal discussions were organized to provide advice for the remaining course of the pregnancy, and information on peri-natal care and assistance was given. General information was also given on functional and aesthetic sequelae, difficulties in language development, the nature and schedule of the various surgical procedures that would or might be indicated.^23^ Later, discussions were organized with the children and their parents to provide support if, and whenever, the child/adolescent experienced periods of anxiety, poor self-esteem, or even depression. Psychological support was offered until the age of 20, depending on the child's and/or the family's need. The psychologist was present at all multidisciplinary consultations. The psychological consultation offered by specialists in the course of each pluridisciplinary consultation helps to spot risky situations and to set up a program of support adapted to each individual child.

### Secondary Surgery

*Oro-nasal fistula closure*: all oro-nasal fistulas were closed before the children were 5 years old if they, or their parents, expressed a social discomfort due to fluids coming through the nose (“jetage”) or in case of speech repercussion. The technique for the closure of the fistula was based on the elevation of the mucoperiosteum of the entire palate. Closure of the fistula was postponed as long as possible, in order to minimize adverse consequences on the growth of the maxilla.

*Secondary labioplasty:* secondary lip surgery in uCLP children was normally done if the cupid's bow was not perfect or if the height of the repaired lip was too short. The height of the lip was determined by using a caliper to measure the height of the outer border on the vertical line from the outer alar base. In most cases, the repaired lip was too short and a Z-plasty was done by opening the skin from the lip to the nostril.

*Rhinoplasty: an early operation* consisted in repositioning the alar cartilage through an incision inside the nostril. A thermoformable splint was used for several days or weeks. Late rhinoplasty or septorhinoplasty was performed when the nose was fully grown. Both objective and subjective indications were considered. The operation involved a small incision below the nose and necessitated a cartilage graft taken from the ear. The deviation of the nasal septum was also corrected during the same surgery. A thermoformable splint may also have been necessary for several days or weeks. It represented the final step after years and years of follow-up.

## Results

Seventy-nine files of children born with a cleft (Table 3) were reviewed, of which 34 were retained: 15 right and 19 left complete clefts of the lip and palate. Two children born with chromosomal abnormalities and uCLP were excluded. The 34 children born with uCLP had undergone in our hospital the same surgical procedure at 3 and 5 months of age, performed by the same senior surgeon. All results were recorded at the end of the follow-up, that is, at 18 years of age.

**Table 3.** Results of Speech, Fistula, Pharyngeal Flap, and Hearing Evaluation at 18 Years Are Summarized. Eighty-Eight Children Needed Grommets. Tympanosclerosis Was Present in 11 of our 34 Patients and Number of Alveolar Grafts, Le Fort Surgery, Secondary lip Surgery, Rhinoplasty and Anesthesia Performed Until 18 Years of Age.

	At 18 years of age
Phonation 1	55%
Phonation 1/2	30%
Phonation 2b	15%
Percentage of fistula	60%
Percentage of pharyngeal flaps done	7%
Mean of age	9.5
Median of age	7.5
SD	5.06
Grommets	88%
Mean number/child	1.89
Median number/child	2
SD	1.1
Tympanosclerosis	32% (11)
Percentage of bone graft needed (mean age)	97%
Mean of age	8.57
Median of age	9
SD	2.47
Percentage of Le Fort osteotomy done	20.5%
Mean of age	15.28
Median of age	16
SD	1.11
Secondary surgery for lip	29%
Mean of age	12.8
Median of age	13.5
SD	5.11
Rhinoplasty	29%
Mean of age	14.8
Median of age	12.5
SD	3.5
Mean number of anesthesia	7.1
Mean number	7
Median number	6
SD	2.18
Mean number of multidisciplinary Consultations done since the age of three	5.7

These 34 uCLPs were operated on following the inverse Malek procedure in two stages: at a median age of 3.2 months for the veloplasty, and 5.9 months for the labio and palatoplasty. Sixty per cent had a fistula whose closure required a median of 1.5 operations. Eighty-eight percent (88%) had grommets, of which four required several operations. The mean number of grommets per child was 1.89 with a median of 2 (SD, 1.1). But 4 children required more than 2 operations to re-insert grommets during the follow-up. Eleven children developed tympanoscerosis.

The presence of nasal phonation led to automatic classification as a type 2 phonation score. Based on the phonatory score, 7% of the children were operated on at the age of 6 following the Sanvenero-Rosselli technique for a pharyngeal flap. The mean age was 9.5 years with a median age of 7.5 (SD, 5.06).

Of the 34 children, 55% had a normal phonation with no nasal air emission (phonation 1), 30% a good phonation with intermittent nasal air emission and good intelligibility (phonation 1/2), and 15% a phonation with continuous nasal emission but good intelligibility and no social discomfort (phonation 2b) (Borel-Maisonny classification).

Ninety-seven percent had an alveolar graft at a mean age of 8.5 years with a median of nine (SD, 2.47) and 21% underwent a Le Fort osteotomy at a mean age of 15.2 years with a median of 16 (SD, 1.11).

None of the children required a new operation because of immediate post-operative complications. Over the long-term, one child required a secondary surgical opening of the pharyngeal flap due to symptomatic blockage of the nasal air flow during sports activities; a minor lateral cutback of the pharyngeal flap was performed 6 months after the initial pharyngeal flap in one child and symptoms resolved promptly with no effect on phonation. Evaluation to determine the existence of sleep apnea was done, but no cases were found.

Based on our discussions with the patient, the family, and the team, 29% of the children were operated on for secondary lip surgery, 7 needed surgery on the upper lip to remove excess tissue; and 4 had a realignment of the Cupid's arch (bow). The mean age was 12.8 years with a median age of 13.5 (SD, 5.11). And 29% of the children were operated on for a late rhinoplasty. The mean age was 14.8 years with a median age of 12.5 (SD, 3.5).

A median number of 5.7 multidisciplinary consultations were done. Overall, the mean number of general anesthesias performed was 7 with a median number of 6 (SD, 2.18).

## Discussion

This retrospective study shows that the inverse Malek procedure, with veloplasty at 3 months and palato-labioplasty at 5 months, may require a high number of palatal fistulae but ensures good long-term phonatory results. There is a relatively high percentage of Le Fort I (20.5%) advancement osteotomies combined with the orthodontic treatment at the end of maxillary growth; 29% had a secondary lip procedure and rhinoplasty with a mean number of anesthesia of 7.1.

At birth, a feeding plate was made on the first or second day by the orthodontist of the team in order to facilitate bottle feeding; to maintain the tongue in a normal functional position and thus allow the full growth potential of the maxilla by preventing the tongue from getting into the cleft; and, later, to protect the sutures that closed the palatal cleft after surgery.^24^ However, these goals are not scientifically defined and are the object of controversy among specialists. The size of the plate is adapted to the babies’ maxillary growth on an ongoing basis.

The sequence of operations for children born with an uCLP can vary widely, depending on the local school of surgery and on historical teaching.^25,26^ The inverse Malek procedure adopted in 1990 in our Center was soft palate repair at 3 months and anterior hard palate and lip closure at 5 months.^2^ A vomer-flap was used to reconstruct the nasal layer of the velum. Until 1990, lip closure had been performed at 6 months and complete palate closure at 18 months.

**Figure 5.**
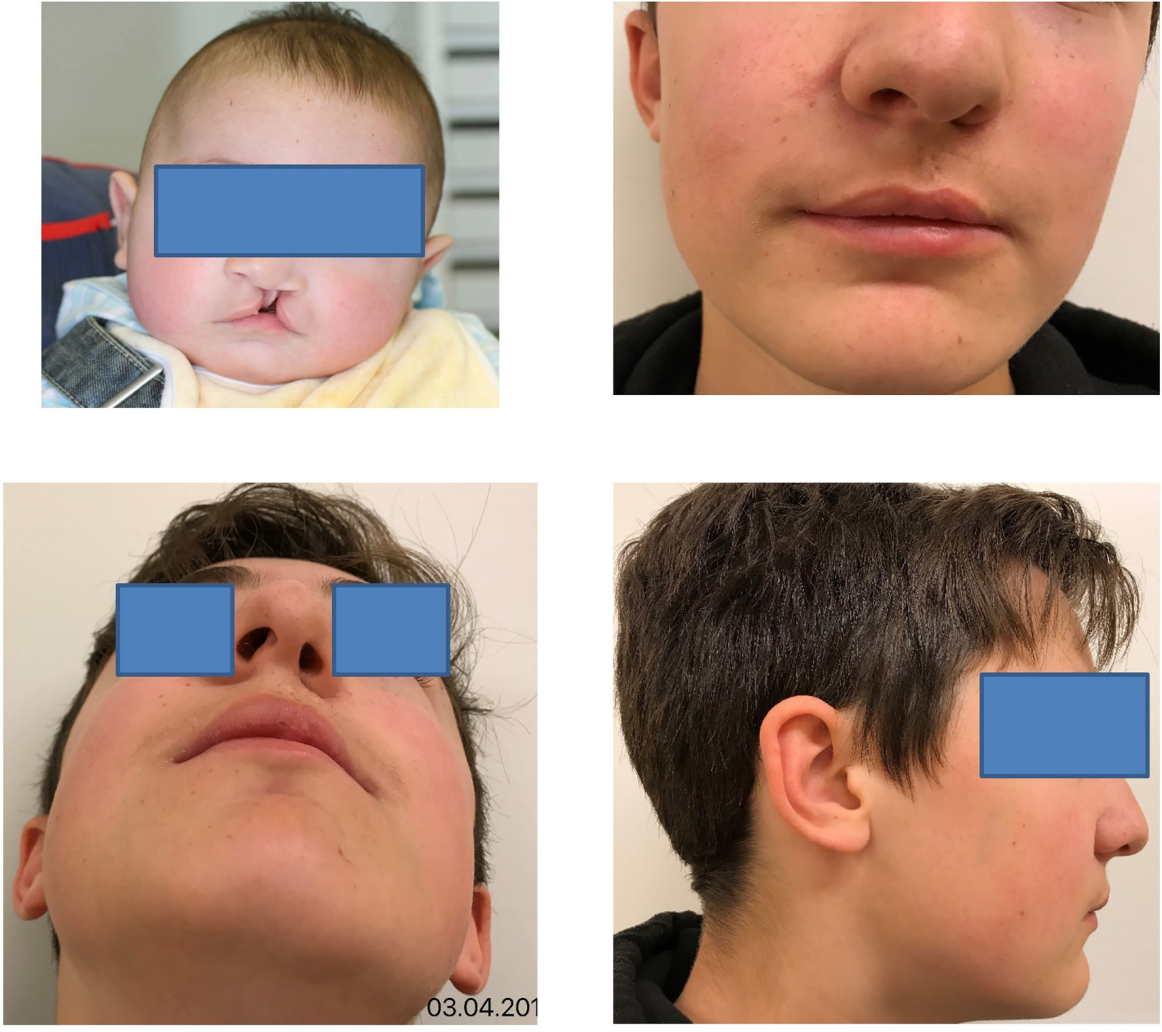
This male patient had left complete cleft lip and palate. He underwent a veloplasty at 3 months and a palatoplasty and a labioplasty following the inverse Malek procedure at 6 months. Speech therapy was begun since 1 year of age with muscle and oral stimulations. A pharyngeal flap was necessary at the age of 7 years of age. Alveolar bone graft was performed at 9 years of age after a short orthodontic treatment. An oro-nasal fistula was closed at 10 years of age for a final phonation of 1b; no surgical Le Fort was performed. Secondary lip surgery was not required but a septorhinoplasty was performed at 15 years of age.

Regarding the lip, one of the surgeon's main concerns is to ensure symmetry between the height of the lip on the cleft side and the normal lip, and surgical procedures are therefore usually based on the principle of Z-plasty (Figures 2 and 5). In the inverse Malek procedure, the exact symmetry between the 2 sides is calculated with mathematical precision. In 1985, Millard presented another version of flaps based on a lateral triangular flap for a unilateral cleft in order to preserve the cupid's bow and the symmetry.^3^

Surgery on the soft palate (veloplasty) should be performed before 18 months of age to facilitate language acquisition.^15,27^ When direct suturing is not possible, it becomes necessary to create a nasal layer using vomerine mucosa, a procedure practiced by Veau and later by Petit.^2,12^ But we must be aware that scars induce maxillary retrusion due to the devascularization of the bone structure after the detachment of the mucoperiosteum and the creation of fibrotic scar tissue. This is even more obvious when surgery is performed in the first months of life. The Furlow technique offers a version of the Z-plasty that makes it possible to lengthen the soft palate, thus ensuring satisfactory maxillary growth, and allowing a good muscular suture which does not impair velar contraction.^28^

Fistulas of the palate remain the major complication of the early primary palate technique. Single-layered closures of the nasal mucosa on the bony vault make fistulas more likely. A second layer of closure on the oral side significantly reduces the incidence of fistulas but does not eliminate them altogether. The best preventive measure is a suture technique based on double-layered closure. When a fistula does exist, there is never spontaneous closure. A first consequence is leakage of fluids or soft food such as milk or water through the nose (when chocolate food falls from the nostril it is known as the “signe du chocolat”); but the passage of food through the nostril can also result from a too short, or scarred soft plate. The second consequence is a speech disorder, called rhinolia, when air comes through the nose during speech. The speech therapist must distinguish between VPI and air coming from the mouth through the fistula. Indication for closure of a fistula depends on the severity of its consequences, but the child must be followed by the speech therapist to make sure that compensatory phenomena due to leakage of air are not present. A technique of closure of the fistula is based on the elevation of the mucoperiosteum of the entire palate. A simple closure of the hole is just not possible. Closure of the fistula must be postponed as long as possible in order to minimize adverse consequences on the growth of the maxilla. In 2015, in order to reduce the number of oro-nasal fistulae, we altered the sequence of operations by closing the total cleft at 4 months. Unfortunately, it is too early to derive any conclusion but we hope to demonstrate a decrease in oro-nasal fistulae.

Many studies have shown that Eustachian tube function in cleft children may remain impaired even after surgery. Secretory medial otitis (OMS) is related to the functional obstruction of the Eustachian tube as a result of anatomical and physiological variations of the tensor and levator palatine muscle.^29^ The presence of secretion in the middle ear or of tympanic membrane retraction/perforation causes difficulties in sound transmission. This impaired anatomical and physiological integrity may therefore hinder the acquisition and development of verbal language.^30^ The use of grommets is largely debated because their indication and the right time to insert them are not clearly defined. Complications of their insertion are numerous, for instance, atelectasis, perforation, tympanic membrane scarring (tympanosclerosis), and cholesteatoma.^31^ An ENT now checks the ears of each of our patients with a microscope during our first operation. Grommets, if needed, are placed during this surgery.

In French-speaking countries, the reference for a perceptual evaluation of VPI, or nasal air emission, is the Borel-Maisonny score (Table 1).^13^ We also use routine nasometry, but we find that its results can be influenced by the child's physical (tired, attentive or not) and/or psychological (intimidated, tensed, or relaxed) state. Video Nasopharyngeal Endoscopy (VNPE) can also be useful and is now the technique of choice in our center; this technique allows direct observation of velopharyngeal movements during speech and could be very useful for the evaluation of the mobility of the palate and the degree of leakage in cases of VPI.^32^ This test can be performed before surgery to provide a reliable surgical indication, or during the anesthesia at the start of the surgery, to provide important information for choosing a flap suited to the individual anatomy, either a simple pharyngeal flap; a ¨push-back” (release of the palate along with and a pharyngeal flap); or a Z-plasty.^33–36^ This investigation was rarely performed on children born between 1997 and 2001; but since 2015, it has been regularly used by our ENT, and it allows a constructive and useful discussion with the surgeon.

We tried to make the cranially based pharyngeal flap as large as possible in order to close the donor site directly and because we know that this flap will narrow in the following months. The variation in the degree of shrinkage of the flap and in the contraction of the scar makes it difficult to accurately predict the final size of the lateral velopharyngeal apertures. It is only in 2010 that VNPE was introduced routinely in our center in the systematic pre-surgery work-up, and this is why most of the children operated on before this date lacked this important aspect of their evaluation. We think that VNPE should always be performed because it provides important information for tailoring the pharyngeal flap to the individual anatomy.

In our study, one child needed a second pharyngeal flap operation due to flap dehiscence, and one other child needed a secondary enlargement of one lateral port due to troublesome unilateral nasal obstruction. In some of our children being treated for uCLP, sleep apnea syndrome (OSAS) was identified by clinical signs of respiratory obstruction, snoring, or morning fatigue. Since 2015, we perform polysomnography of all children before pharyngoplasty, and again 6 months after the operation. It is very important to schedule this examination before any pharyngoplasty because OSAS is a contraindication to pharyngeal surgery.

Since 2002, we have organized speech therapy workshops for parents and their children (twice a year, 2-hour sessions with 4-5 patients of the same age and presenting the same cleft type) where the speech therapist shows the parents how to work with their children at home (guidance) in order to strengthen the velopharyngeal muscle complex.

Indications for orthodontic treatment are both functional and aesthetic. For us, the first phase of active treatment aims at the normalization of the shape of the upper dental arch by using an asymmetrical expansion plate before consolidation by means of a bone graft.^25^ The aim of the bone graft is to close a cleft in the alveolar process and to allow for subsequent orthodontic space closure. The second phase of the orthodontic treatment starts after the complete healing of the bone graft (autograft). The aligned teeth are stabilized by a retainer made of fine steel wire bonded to the lingual side of the teeth with composite resin. The third phase starts around the age of 12. Intermaxillary dental relationships and teeth malpositions will be corrected. In a percentage of cases, a purely orthodontic approach may be insufficient, and orthognathic surgery may have to be considered. Excellent dental hygiene is essential throughout the treatment and beyond, and is another of our priorities.

The planification of the alveolar bone graft must be well thought out, even if its exact timing cannot always be precisely determined and varies largely between children. We usually organize the bone graft around 9 years of age in our center, and we use cancellous bone harvested in the mandibular ramus area, distal to the wisdom tooth area.

When planning orthognathic surgery, many factors, such as facial profile harmony and aesthetics, intermaxillary discrepancies and dento-alveolar relationship, are taken into account. Ross^37^ compared lateral cephalograms of uCLP children whose cleft surgery had relied on different techniques: he showed very similar results and measurements between children whose clefts were closed according to the inverse Malek procedure, and children whose clefts were closed with a conventional technique. However appropriate primary surgical procedures may be, indications for orthognathic surgery may be found in the later stages of the patient's development. The Le Fort I advancement osteotomy is most often considered, and sometimes mandibular adaptation surgery may be an additional option. Besides, in cases of a transversal collapse of the maxillary arch, essentially caused by the presence of scar tissue, maxillary advancement cannot always be achieved in one piece. In these cases, the maxilla needs to be segmented in 2 or, more rarely, in 3 pieces. The frequency of indications in the literature for a Le Fort I osteotomy in uCLP varies from 22% to 48.3%.^38^ These differences may arise from different management protocols, but also depend on the patient's access to adequate pre-surgical orthodontic care. The criteria used to determine the need for orthognathic surgery are also subjective to some extent, and therefore may vary between surgical teams.

Results in terms of growth and aesthetic success are still being evaluated^39,40^ But rhinoplasty is planned ideally at the end of the growth. The operation involves a small incision below the nose and necessitates a cartilage graft taken from the ear to lengthen the columella.^41^ The deviation of the nasal septum is also corrected during the same procedure. None of the children in our study had early rhinoplasty, but all children needed a rhinoplasty at 15 years. We therefore, in 2015, began doing early rhinoplasties, at 4 months of age, with section of the septum, alar dissection and use of a removable splint for 2 months (Table 4).

**Table 4.** Differences of the Protocol Established from 1996 to 2001 and the Actual Protocol.

	1996–2001	actual
**Time of primary cleft palate or lip repairs**	Three and six months	Four months
**Rhinoplasty**	At the end of the follow-up during adolescence	Primary rhinoplasty at 4 months
**Myringotomy**	At 6 months, from case to case	At 4 months with a microscope
**Polysomnography**	Clinical evaluation and polysomnography if needed	Polysomnography before and after a pharyngeal flap
**Superior Pharnygeal flap**	Yes	With a push-back
**Speech therapy workshop**	No	Since 2002
**Bone graft before at 5 to 6 years**	No	In discussion

Prenatal discussions provide advice for the remaining course of the pregnancy and also give the parents information on perinatal care and assistance. A birth in itself modifies the family harmony, and the arrival of a child causes psychological and physiological upheavals that specialists define as periods of crisis in maturation. After the diagnosis of a cleft, the parents are overcome with worry and anxiety. Certain parents develop feelings of culpability, of loss of control, of inadequacy as genitors, feelings which could well have an incidence on the psycho-affective development of the child.^23^ The social skills of the child himself, and later of the adolescent, may also be affected, with resulting manifestations of anxiety, poor self-esteem, or depression. The child, or adolescent, will be confronted with difficult episodes at different times of his life, for instance and to name only a few, the start of school, the relation with peers, puberty, and the relationship with girl- or boy-friends. The psychological consultation offered by specialists in the course of each pluridisciplinary consultation helps to spot risky situations and to set up a program of support adapted to each individual child. The real success of 20 years of work and follow-up should be assessed by the patient himself (Figure 5); we therefore decided to create a questionnaire on aesthetic and functional satisfaction, submitted to the patient when he reaches the age of 18. We submitted this same questionnaire to the members of the medical team, and it will be interesting to compare the results.

## Conclusions

The results of our retrospective study are based on one surgical protocol of primary surgery performed by one single experienced surgeon in our institution on children born with uCLP. The inverse Malek procedure is related to a high number of palatal fistulae at the origin of embarrassing rhinolalia and therefore may require a second operation for palatal closure. On the other hand, the results of the closure of the soft palate are good, with no long-term 2 M or 2/3 phonation due to a VPI: a one-time operation is currently being carried out but the overall consequences will only be apparent at the age of 18. The possible need for grommets is currently determined during the operation at 4 months of age, with a microscope to verify the presence of an OMS. There is a relatively high percentage of indications for a Le Fort I (22%) advancement osteotomy: this surgical procedure is therefore clearly explained to parents in early consultations as being part of the ongoing treatment and not the result of a failure in the follow-up. Operating children efficiently while respecting the dental articulation will reduce the need for possible later orthodontic care. Since 29% had a secondary rhinoplasty at 15 years of age, we currently perform primary rhinoplasty during the first operation.

Finally, the number of children (34) remaining after the exclusion criteria were applied (79 files of children were reviewed) is not high. But the same surgical procedure, performed at 3 and 5 months of age, in our hospital, and by the same surgeon, showed comparable results in all 34 children.
